# Elevated Glycemic Gap Predicts Acute Respiratory Failure and In-hospital Mortality in Acute Heart Failure Patients with Diabetes

**DOI:** 10.1038/s41598-019-42666-0

**Published:** 2019-04-18

**Authors:** Wen-I Liao, Jen-Chun Wang, Chin-Sheng Lin, Chih-Jen Yang, Chia-Ching Hsu, Shi-Jye Chu, Chi-Ming Chu, Shih-Hung Tsai

**Affiliations:** 1 Department of Emergency Medicine, Tri-Service General Hospital, National Defense Medical Center, Taipei, Taiwan; 2 Division of Cardiology, Department of Internal Medicine, Tri-Service General Hospital, National Defense Medical Center, Taipei, Taiwan; 3 Department of Internal Medicine, Tri-Service General Hospital, National Defense Medical Center, Taipei, Taiwan; 4 School of Public Health, National Defense Medical Center, Taipei, Taiwan; 5 Department of Physiology and Biophysics, Graduate Institute of Physiology, National Defense Medical Center, Taipei, Taiwan

**Keywords:** Cardiology, Heart failure, Endocrinology

## Abstract

Diabetes is a common comorbidity in patients hospitalized for acute heart failure (AHF), but the relationship between admission glucose level, glycemic gap, and in-hospital mortality in patients with both conditions has not been investigated thoroughly. Clinical data for admission glucose, glycemic gap and in-hospital death in 425 diabetic patients hospitalized because of AHF were collected retrospectively. Glycemic gap was calculated as the A1c-derived average glucose subtracted from the admission plasma glucose level. Receiver operating characteristic (ROC) curves were used to determine the optimal cutoff value for glycemic gap to predict all-cause mortality. Patients with glycemic gap levels >43 mg/dL had higher rates of all-cause death (adjusted hazard ratio, 7.225, 95% confidence interval, 1.355–38.520) than those with glycemic gap levels ≤43 mg/dL. The B-type natriuretic peptide levels incorporated with glycemic gap could increase the predictive capacity for in-hospital mortality and increase the area under the ROC from 0.764 to 0.805 (net reclassification improvement = 9.9%, p < 0.05). In conclusion, glycemic gap may be considered a useful parameter for predicting the disease severity and prognosis of patients with diabetes hospitalized for AHF.

## Introduction

Diabetes is a common comorbidity in acute heart failure (AHF) patients and accounts for 30–40% of the overall population^1,2^. Diabetes may cause cardiac dysfunction in the absence of overt macrovascular disease, the formation of atherosclerosis and hypertension, and complications in response to therapy^1,3^. Hyperglycemia is a common problem in patients with AHF and is an independent risk factor of short-term mortality in non-diabetic patients but not in patients with diabetes^4,5^. Several studies suggest that hyperglycemia was associated with more severe symptoms and worse New York Heart Association (NYHA) functional classification and that in patients with AHF, especially those with previously diagnosed diabetes, hyperglycemia was related to higher in-hospital fatality^6,7^. However, the relationship between admission hyperglycemia and mortality in diabetic patients with AHF or critical illness remains controversial^2,8,9^. Those discrepancies could be partly attributed to long-term glycemic control in diabetic patients. Critical illness can induce stress-induced hyperglycemia (SIH), which results from the excess release of counter-regulatory hormones and anti-inflammatory cytokines, leading to augmented gluconeogenesis and insulin resistance^10–13^. In diabetic patients with acute illness, the phenomenon of admission hyperglycemia could be connected with acute physiological stress, poorer glycemic controls, or a combination of both^14^.

It is necessary to think about background glycemic control when exploring the relationships between admission hyperglycemia and clinical outcomes. Recent studies have shown that hemoglobin A1c (HbA1c)-based adjusted glycemic variables, including glycemic gap and stress hyperglycemia ratio, were linked to severity of disease and unfavorable prognosis in diabetic patients with some infectious diseases, cardiovascular diseases and critical illness^15–21^.

We hypothesized that a higher glycemic gap would be linked to unfavorable outcomes in diabetic patients with AHF. The present investigation was aimed at assessing the relationships between glycemic gap and adverse clinical outcomes in diabetic patients who were hospitalized due to AHF.

## Methods

### Study participants

The use of the clinical data for this study was approved by Institutional Review Board of Tri-Service General Hospital, National Defense Medical Center and waived the requirement for informed consent. This retrospective observational study pooled data of all patients with diabetes hospitalized for AHF between June 1, 2011 and December 31, 2017. We searched the International Classification of Diseases (9^th^ revision) codes 428.0–9 and 250.2–8 to identify the patients with diabetes and AHF. The patients’ medical records were then reviewed manually to enroll only those patients with AHF who had data for admission plasma glucose levels and HbA1c levels within 1 month before or after admission. Diagnosis of diabetes was defined if a patient was discharged from a hospital with a diagnosis of diabetes and/or at least 1 prescription for insulin or an oral antidiabetic agent and/or had an HbA1c level ≥6.5% in the preceding 2 months^22,23^.

Clinical diagnosis of AHF was made according to the presence of classic clinical history and physical examination, i.e., rapid-onset dyspnea and fatigue possibly leading to pulmonary and/or splanchnic congestion and/or peripheral edema, with further confirmation by laboratory measurement of B-type natriuretic peptide (BNP) ≥100 pg/ml, 12-lead electrocardiogram, chest radiography or echocardiography^24,25^. All included patients were admitted to the cardiology ward or intensive care unit (ICU) and were treated with intravenous therapy (inotropes, vasodilators, or diuretics). Patients were excluded based on the following criteria: age ≤18 years, hypoglycemia (plasma glucose <70 mg/dL) at initial presentation, or an admission diagnosis of diabetic ketoacidosis or hyperosmolar hyperglycemic state. Patients with hemoglobin variants (e.g., sickle cell anemia, thalassemia) or with hematologic conditions (e.g., hemolytic anemia) that could interfere with the accuracy of the HbA1c assay were also excluded.

### Study design

We retrospectively reviewed patients’ medical records to determine age; sex; underlying comorbidities; etiology of AHF; current left ventricular ejection fraction (LVEF); laboratory data, including plasma glucose level at initial presentation and HbA1c levels (measured within 1 month before or after admission); and stays in the ICU and hospital. The NYHA functional class was used to assess the severity of AHF, and all the included patients were class III or IV at admission. Ejection fraction (EF) was measured by conventional transthoracic echocardiography according to international standard criteria. Cardiogenic shock was defined as severe hypotension of an acute cardiac cause despite adequate filling status (need for vasopressor therapy to maintain systolic blood pressure ≥90 mmHg). Acute respiratory failure (ARF) was defined as a patient with respiratory distress requiring noninvasive positive pressure ventilation (NIPPV) or endotracheal intubation with mechanical ventilation; acute kidney injury (AKI) was defined as serum creatinine elevated >0.3 mg/dL or 50% from baseline^26^; and upper gastrointestinal bleeding (UGIB) was defined as melena with positive occult blood examination, bright-red blood discharged from the nasogastric tube, or endoscopic evidence of mucosal bleeding. The primary endpoints were all-cause mortality during index hospitalization. Cardiovascular mortality was a composite of cardiac death attributed to cardiogenic shock, ventricular arrhythmia or myocardial infarction.

### Measurements of admission glucose, HbA1c, and HbA1c-based adjusted glycemic gap

The admission glucose level was defined as initial plasma glucose levels upon admission to the emergency department. HbA1c was measured using high-performance liquid chromatography on a blood analyzer (Primus CLC 385; Primus Corporation, Kansas City, MO, USA). The long-term average glucose levels can be derived from the HbA1c values by using an equation from an international multicenter study of HbA1c-derived average glucose (ADAG). The estimated chronic average glucose levels (eAG) over the past 3 months was calculated from the equation AG = 28.7 × HbA1c − 46.7^27^. Glycemic gap was calculated as the ADAG level subtracted from the admission glucose level.

### Statistical analysis

Continuous data are expressed as the means ± standard deviations and were analyzed using the two-tailed Student’s *t* test. Categorical data are expressed as frequencies (%) and were compared using the chi-square test or Fisher’s exact test. A receiver operating characteristic (ROC) curve was plotted to calculate the area under the ROC (AUROC). Analyses were performed on Statistical Package for the Social Sciences version 22.0 statistical software (IBM Corp., Armonk, NY, USA), and differences with p values < 0.05 were considered statistically significant. Multivariate Cox proportional hazard models were applied to determine independent predictors of in-hospital all-cause mortality for AHF. The covariates included in the multivariable regression models were chosen as potential confounding factors based on their significance in univariable analyses. The log-rank test was performed to determine the statistical significance of the effects of all-cause mortality and cardiovascular mortality on Kaplan–Meier survival curves. The net reclassification improvement (NRI), a function of MATLAB (MathWorks, Natick, MA, USA), was performed to evaluate the improvement in overall predictive capacity after adding parameters^28^.

## Results

### Patient study population and clinical outcomes

We initially enrolled 425 hospitalized patients with diabetes and AHF. Patients were eliminated owning to admission hypoglycemia (n = 30). Ultimately, we identified 395 patients after a detailed medical record review. Of these patients, 46.3% were men, 7.1% died during hospitalization, 43.3% were admitted to the ICU, 6.3% experienced cardiogenic shock, 20.8% experienced ARF, 17.0% experienced AKI, and 5.1% developed UGIB. A comparison of the baseline characteristics of the enrolled patients between survivors and nonsurvivors during hospitalization is shown in Table 1. The glycemic gap levels were significantly higher in nonsurviving patients (109.6 ± 68.1 mg/dL) than in surviving patients (49.3 ± 96.8 mg/dL). The admission glucose levels were 262.2 ± 94.1 mg/dL in nonsurvivors and 221.5 ± 107.4 mg/dL in survivors, without a statistically significant difference. The mean ages were significantly older in nonsurvivors (83.3 ± 12.6 years) than in survivors (75.1 ± 12.2 years). Nonsurvivors had a greater likelihood of ischemic etiology of AHF, prior stroke and peripheral arterial occlusive disease than survivors. Moreover, nonsurvivors had higher levels of BNP and aspartate aminotransferase (AST) and lower levels of albumin than survivors and were more likely to develop cardiogenic shock, ventricular fibrillation (VF) or ventricular tachycardia (VT), ARF and UGIB than survivors.

**Table 1:** Comparison of the characteristics of the survivors and nonsurvivors.

Baseline characteristics	Survivors (n = 367)	Nonsurvivors (n = 28)	*p* value
Age (yrs)	75.1 ± 12.2	83.3 ± 12.6	0.001*
Male	172 (46.9%)	11 (39.3%)	0.44
Hypertension	320 (87.2%)	22 (78.6%)	0.20
Dyslipidemia	173 (47.1%)	10 (35.7%)	0.24
Coronary artery disease	281 (76.8%)	22 (78.6%)	0.83
Peripheral arterial occlusive disease	34 (9.3%)	6 (21.4%)	0.04*
Prior stroke	30 (8.2%)	8 (28.6%)	<0.001*
Chronic kidney disease	218 (59.6%)	18 (64.3%)	0.62
History of atrial fibrillation	101 (27.5%)	6 (21.4%)	0.48
Chronic obstructive pulmonary disease	22 (6.0%)	4 (14.3%)	0.09
Current left ventricular ejection fraction	45.0 ± 17.7%	39.8 ± 14.6%	0.16
NYHA functional classification			0.051
III	201 (54.8%)	10 (35.7%)	
IV	166 (45.2%)	18 (64.3%)	
**Etiology of heart failure**			
Valvular heart disease	86 (23.4%)	5 (17.9%)	0.50
Ischemic heart disease	117 (31.9%)	16 (57.1%)	0.006*
Idiopathic dilated cardiomyopathy	28 (7.6%)	1 (3.2%)	0.43
Hypertensive heart disease	49 (13.4%)	2 (7.1%)	0.35
Atrial fibrillation	23 (6.3%)	1 (3.6%)	0.57
Others	62 (16.9%)	3 (10.7%)	0.40
**Laboratory findings at admission**			
Glycemic gap, mg/dL	49.3 ± 96.8	109.6 ± 68.1	0.001*
Admission glucose, mg/dL	221.5 ± 107.4	262.2 ± 94.1	0.052
Max. glucose during first 48 h, mg/dL	269.5 ± 103.9	307.1 ± 110.4	0.07
HbA1c, %	7.6 ± 1.8	6.9 ± 1.7	0.06
BNP, pg/ml	1390 ± 1286	2639 ± 1517	<0.001*
Hb, g/dL	11.0 ± 2.3	11.0 ± 2.5	0.96
Cr, mg/dL	2.9 ± 2.4	3.6 ± 3.4	0.13
Sodium, mmol/L	135.0 ± 5.2	134.9 ± 11.0	0.95
AST, U/L	37.8 ± 82.1	132.0 ± 181.8	<0.001*
Albumin, g/dL	3.2 ± 0.4	2.8 ± 0.6	<0.001*
Total bilirubin, mg/dL	0.7 ± 0.5	0.8 ± 0.3	0.70
Total cholesterol, mg/dL	152.6 ± 46.0	150.8 ± 49.9	0.88
Triglyceride, mg/dL	122.6 ± 89.0	120.4 ± 58.2	0.92
HDL, mg/dL	39.3 ± 17.5	46.0 ± 15.6	0.60
LDL, mg/dL	94.7 ± 40.2	110.8 ± 65.0	0.35
Uric acid, mg/dL	7.5 ± 2.7	8.1 ± 2.9	0.39
TSH, µIU/ml	2.9 ± 4.0	1.0 ± 1.1	0.30
**Clinical outcome**			
Cardiogenic shock	11 (3%)	14 (50%)	<0.001*
VF, VT	4 (1.1%)	4 (14.3%)	<0.001*
Acute respiratory failure	55 (15.0%)	27 (96.4%)	<0.001*
UGIB	14 (3.8%)	6 (21.4%)	<0.001*
Acute kidney injury	60 (16.3%)	7 (25.0%)	0.24
ICU admission	151 (41.1%)	20 (71.4%)	0.002*
ICU hospitalization (days)	3.5 ± 9.2	11.6 ± 16.3	<0.001*
Total hospitalization (days)	11.5 ± 12.4	18.7 ± 18.7	0.005*

*Continuous data are expressed as the mean*s ± *standard deviation*s, and categorical data are expressed as frequencies (%). *P < 0.05.

NYHA, New York Heart Association functional class; Max., maximum; HbA1c, hemoglobin A1c; BNP, brain natriuretic peptide; AST, aspartate aminotransferase; HDL, high*-density lipoprotein* cholesterol; LDL, *low-density lipoprotein* cholesterol; TSH, thyroid stimulating hormone; VF, ventricular fibrillation; VT, ventricular tachycardia; UGIB, Upper gastrointestinal bleeding; ICU, Intensive care unit.

### Association between glycemic gap and heart failure (HF)

**Figure 1:**
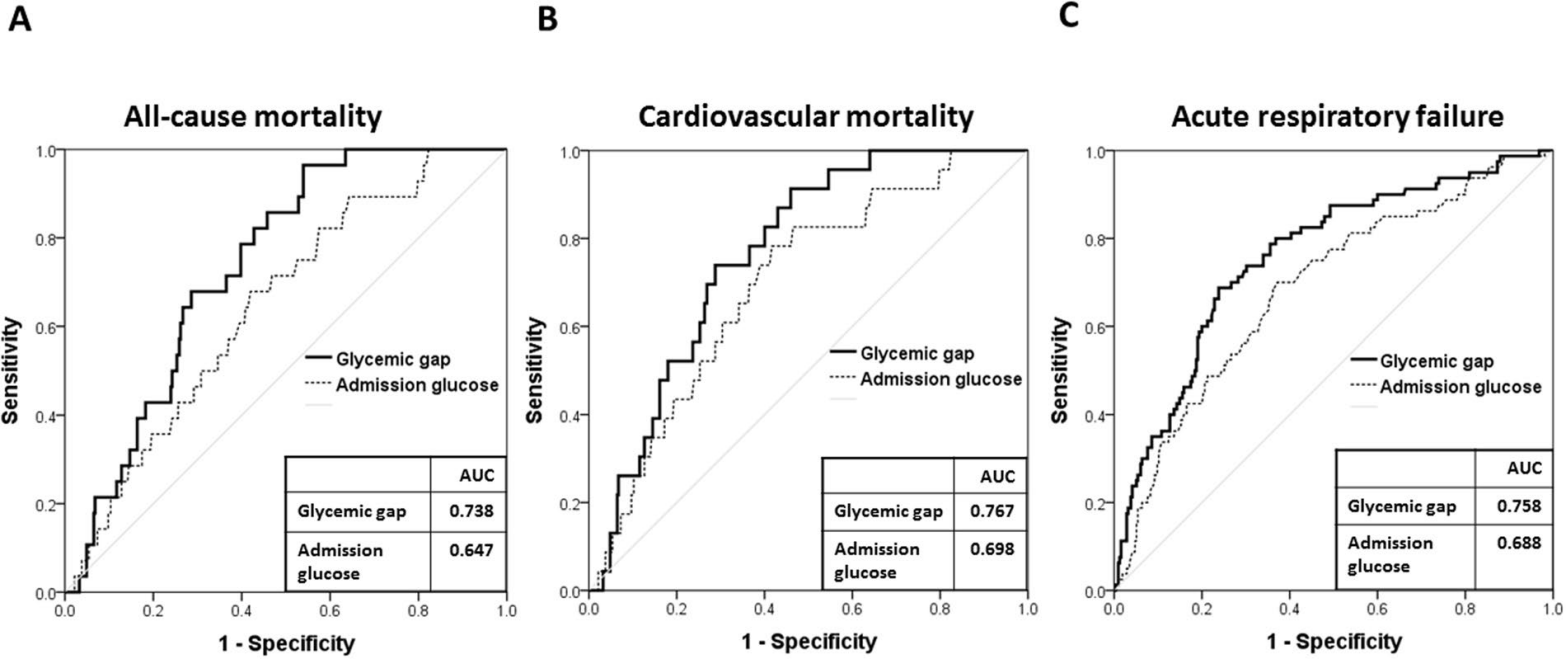
ROC of admission glucose and glycemic gap to predict all-cause mortality (**A**), cardiovascular mortality (**B**) and acute respiratory failure (**C**) AUC: Area under the curve; ROC: Receiver operating characteristic.

We performed a ROC analysis to determine the cutoff values of glycemic gap to maximize the predictive value for the incidence rates of all-cause and cardiovascular mortality. In both analyses, the optimal cutoff value of the glycemic gap was 43 mg/dL, with maximum AUROC of 0.738 (95% CI = 0.667–0.809) (sensitivity 85.7% and specificity 54.2%) in all-cause mortality, 0.767 (95% CI = 0.692–0.842) (sensitivity 91.3% and specificity 54.0%) in cardiovascular mortality and 0.758 (95% CI = 0.699–0.818) (sensitivity 81.3% and specificity 59.7%) in ARF. Moreover, glycemic gap produced higher AUROC values than did admission glucose for predicting all-cause mortality, cardiovascular mortality and ARF (Fig. 1).

**Figure 2:**
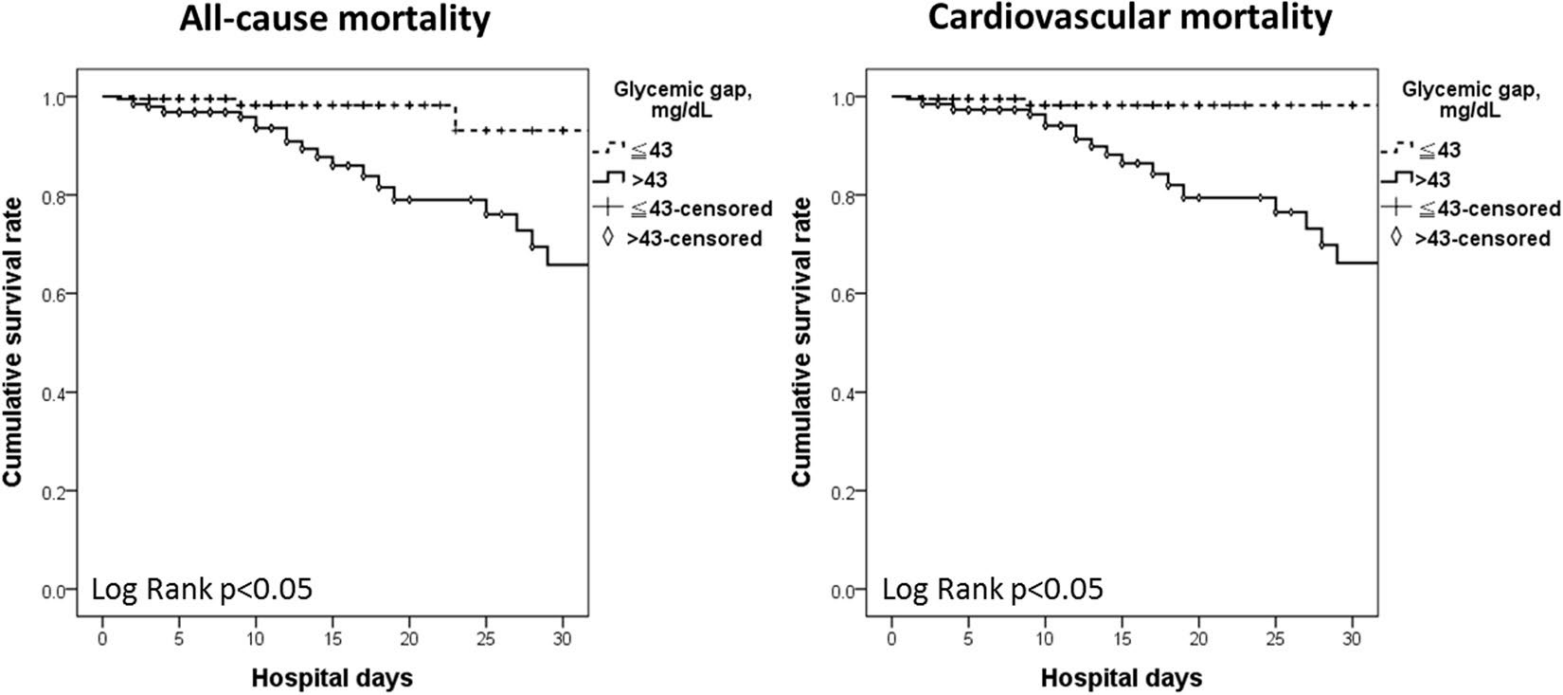
Kaplan–Meier survival curves for all-cause mortality and cardiovascular mortality.

Compared with patients with a glycemic gap level of ≤43 mg/dL, patients with a glycemic gap level of >43 mg/dL had a significantly increased risk of all-cause mortality, cardiovascular mortality, cardiogenic shock, ARF, and ICU admission with prolonged ICU and total hospital stays (Table 2**)**. A Kaplan–Meier survival curve revealed that all-cause and cardiovascular mortality rates during hospitalization had significantly higher associations with a glycemic gap level >43 mg/dL than with a glycemic gap level ≤43 mg/dL (log-rank test, p < 0.05, Fig. 2). In a univariate Cox proportional hazards regression analysis, patients with a glycemic gap level >43 mg/dL [hazard ratio (HR), 4.568; 95% confidence interval (CI), 1.574–13.258; p = 0.005] had significantly increased in-hospital mortality but not admission glucose. In addition, increased age (HR, 1.048; 95% CI, 1.010–1.087; p = 0.01), prior stroke (HR, 2.744; 95% CI, 1.182–6.369; p = 0.02), greater log BNP level (HR, 6.299; 95% CI, 1.725–22.999; p = 0.005), higher AST level (HR, 1.001; 95% CI, 1.000–1.003; p = 0.04), and lower albumin level (HR, 0.344; 95% CI, 0.166–0.715; p = 0.004) showed values with p < 0.05 in univariate analyses and were included in the multivariate analysis. In multivariate Cox proportional hazards regression, patients with a glycemic gap >43 mg/dL presented a 7.22-fold greater risk of in-hospital mortality than those with a gap ≤43 mg/dL after adjusting for age, sex, prior stroke and levels of log BNP, AST and albumin. In addition to elevated glycemic gap levels, increased age, greater log BNP levels and lower albumin levels independently predicted in-hospital mortality in multivariate analyses (Table 3).

**Table 2:** Clinical outcome versus glycemic gap of patients with both diabetes and acute decompensated congestive heart failure.

	Glycemic gap $\leqq$ 43 mg/dL (n = 202)	Glycemic gap > 43 mg/dL (n = 193)	*p* value
All-cause mortality	4 (2.0%)	24 (12.4%)	<0.001*
Cardiovascular mortality	2 (1.0%)	21 (10.9%)	<0.001*
Cardiogenic shock	4 (2.0%)	21 (10.9%)	0.001*
VF, VT	2 (1.0%)	6 (3.1%)	0.14
Acute respiratory failure	15 (8.4%)	65 (33.7%)	<0.001*
UGIB	10 (5.0%)	10 (5.2%)	0.92
Acute kidney injury	33 (16.3%)	34 (17.6%)	0.74
ICU admission	72 (35.6%)	99 (51.3%)	0.002*
ICU hospitalization (days)	3.0 ± 6.9	5.1 ± 12.5	0.04*
Total hospitalization (days)	10.5 ± 10.5	13.6 ± 15.2	0.02*
NYHA functional classification			0.99
III	108(53.5%)	103(53.4%)	
IV	94(46.5%)	90(46.6%)	

*p < 0.05.

VF, ventricular fibrillation; VT, ventricular tachycardia; UGIB, upper gastrointestinal bleeding; ICU, intensive care unit; NYHA, New York Heart Association functional class.

**Table 3:** Univariate and multivariate Cox hazard regression analyses for the development of all-cause mortality in patients with diabetes and acute decompensated heart failure.

	Univariate		Multivariate	
	HR (95% CI)	*p-*value	HR (95% CI)	*p-*value
Age	1.048 (1.010–1.087)	0.01^*^	1.055 (1.000–1.113)	0.049^*^
Male	0.741 (0.346–1.587)	0.44	0.735 (0.235–2.298)	0.60
Hypertension	0.564 (0.226–1.405)	0.22		
CAD	1.011 (0.408–2.507)	0.98		
Prior stroke	2.744 (1.182–6.369)	0.02^*^	1.056 (0.358–3.120)	0.92
PAOD	1.246 (0.496–3.126)	0.64		
CKD	1.145 (0.524–2.502)	0.73		
History of Af	0.669 (0.270–1.657)	0.39		
History of VHD	0.870 (0.393–1.930)	0.73		
COPD	1.861 (0.641–5.405)	0.25		
Current LVEF	0.994 (0.970–1.018)	0.63		
Glycemic gaps		0.005^*^		0.02^*^
Gap ≤43 mg/dL	1		1	
Elevated gap >43 mg/dL	4.568 (1.574–13.258)		7.225 (1.355–38.520)	
Log BNP	6.299 (1.725–22.999)	0.005^*^	51.69 (4.978–536.752)	0.001^*^
Admission glucose	1.003 (1.000–1.005)	0.08		
HbA1c	0.872 (0.652–1.165)	0.35		
Hb	0.982 (0.836–1.154)	0.83		
Sodium	0.987 (0.940–1.035)	0.59		
Cr	1.082 (0.942–1.244)	0.26		
AST	1.001 (1.000–1.003)	0.04^*^	1.001 (0.997–1.004)	0.63
Albumin	0.344 (0.166–0.715)	0.004^*^	0.220 (0.077–0.633)	0.005^*^
Uric acid	1.072 (0.913–1.258)	0.40		
Total cholesterol	0.999 (0.988–1.010)	0.88		
Triglyceride	1.000 (0.994–1.006)	0.92		
HDL	1.018 (0.954–1.086)	0.60		
LDL	1.008 (0.991–1.025)	0.36		

CI, confidence interval; HR, hazard ratio; CAD, coronary artery disease; PAOD, peripheral arterial occlusive disease; CKD, chronic kidney disease; Af, atrial fibrillation; VHD, valvular heart disease; COPD, chronic obstructive pulmonary disease; LVEF; current left ventricular ejection fraction; BNP, brain natriuretic peptide; HbA1c, hemoglobin A1c; AST, aspartate aminotransferase; HDL, high-density lipoprotein cholesterol; LDL, low-density lipoprotein cholesterol. ^*^p < 0.05.

### Correlation between glycemic gap and BNP

**Figure 3:**
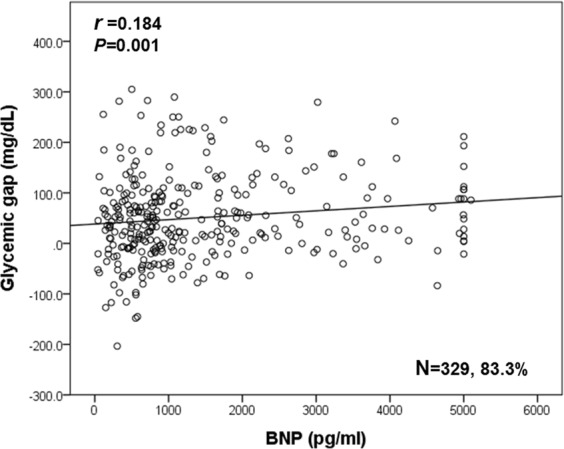
Correlations between glycemic gap and BNP levels.

**Figure 4:**
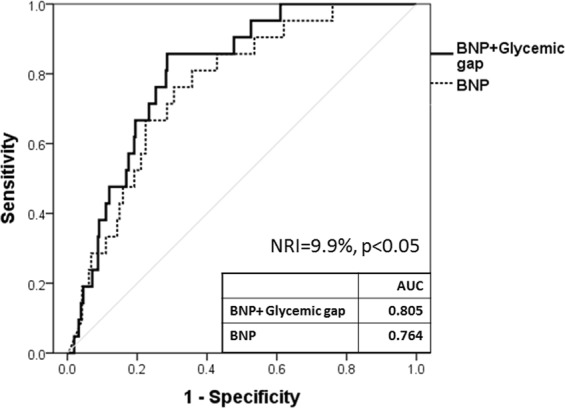
Effects of integrating glycemic gap with BNP levels.

The correlations were statically significant but weak between glycemic gap and BNP levels (r = 0.184, p < 0.05, Fig. 3). Incorporating glycemic gap with BNP increased the discriminative performance of BNP for predicting all-cause mortality and raised the AUROC from 0.764 (95% CI = 0.673– 0.854) to 0.805 (95% CI = 0.729– 0.880) (NRI = 9.9%, P < 0.05, Fig. 4).

## Discussion

Our data indicated that 1) compared to acute hyperglycemia and HbA1c, glycemic gap had greater discriminative power for predicting in-hospital mortality and the development of ARF in patients with diabetes admitted to the hospital due to AHF, and 2) incorporating glycemic gap could further increase the discriminative power of BNP, thus strengthening the clinical significance of glycemic gap in AHF.

Studies regarding admission glucose and mortality in patients hospitalized with AHF and diabetes are still controversial^8,29^. Some studies have indicated that SIH is linked to unfavorable clinical outcomes in a variety of critical illnesses^14,30–32^. A previous study also indicated that acute fluctuations in glucose concentrations are related to elevated mortality in acutely ill patients, independent of mean glucose concentration^33^. Patients without diabetes who died during the hospitalization period for AHF had, on average, higher first plasma glucose values than those who survived^5^. However, several studies have reported that the association between SIH and severity of disease is relatively slight in diabetic patients with critical illness^34–37^. The association between acute hyperglycemia, in terms of time-weighted glucose levels, and ICU mortality was stronger in patients without diabetes than in patients with diabetes^38^. A recent study showed that the maximum glucose levels in the first 24 h of ICU admission did not predict hospital fatality in a medical ICU^39^. These discordant findings led to the question of whether hyperglycemia contributes to increased mortality or is merely a marker of increased illness severity. Patients with diabetes presenting with acute illness could have a spectrum of premorbid glycemic control, from satisfactory to poor^13^. In patients with diabetes, acute hyperglycemia at admission could be the result of SIH, chronic poor glucose control or a combination of both. HbA1c levels are useful for assessing long-term overall glycemic control over the preceding 2–3 months, as they have characteristics of lesser biological variability and are relatively not interfered by acute stress^23^. By using HbA1c-adjusted glycemic variables, we were able to get rid of the possible interference of long-term hyperglycemia in patients with diabetes. In the current study, glycemic gap rather than admission glucose levels predicted hospital fatality in patients with diabetes and AHF. Similar findings were disclosed in patients with diabetes-sustained liver abscess^15^, community-acquired pneumonia^16^, ICU mortalities^17^, acute myocardial infarction (AMI)^19^, acute ischemic stroke^21^ and chronic obstructive lung disease with acute exacerbation^20^. The use of glycemic gap could explain the “diabetes paradox” and controversies about the association between admission hyperglycemia, chronic glycemic control, and adverse events^40^.

The prognosis for patients with HF is worse in those with diabetes than in those without diabetes, independent of the presence of coronary artery disease or its associated risk factors^41^. However, poorer glycemic control was not correlated with worse clinical outcomes in critically ill patients^33,38,42^. Glycemic control impacts the clinical outcomes in patients with HF and diabetes in a U-shaped relationship with poorer survival at low and high mean HbA1c levels^43^. Optimal chronic glycemic control occurs at a range of HbA1c from 7.1 to 8.0% for patients with diabetes who have HF^43,44^. Among HF patients with LVEF ≤ 45% who were not receiving antihyperglycemic drugs, those with HbA1c > 6.7% had greater mortality than those with HbA1c ≤ 6.7%^45^. Despite these data, studies examining the relationship between HbA1c and clinical outcomes in diabetic patients with HF have also disclosed discrepant results. Eshaghian *et al*. demonstrated that HbA1c levels >7.0 were associated with improved survival in patients with diabetes and advanced HF compared with HbA1c levels ≤7.0^46^. In our study, the HbA1c levels did not correlate with hospital death in diabetic patients with AHF. Further investigation is necessary to determine the relationship between HbA1c and adverse events in diabetic patients with AHF.

Patients who had AHF complicated with ARF could further have SIH. Severe hyperglycemia was significantly correlated with hospital fatality in patients with cardiogenic shock^47^. In the current study, glycemic gap >43 mg/dL had a higher prevalence of ARF and cardiogenic shock than glycemic gap ≤43 mg/dL, further supporting the positive correlation between glycemic gap and severity of AHF. The levels of plasma BNP are closely related to HF severity, and BNP is used as a diagnostic and prognostic tool in both acute and chronic settings by international guidelines^25^. HF patients with diabetes had higher levels of BNP than those without diabetes in the chronic setting but not in the acute care setting^48,49^. BNP levels negatively correlate with systolic and diastolic cardiac function in patients with diabetes^50^. In accordance with our findings, a recent large study demonstrated that BNP levels and age were the strongest predictors of death in patients with HF^51^. We hypothesized that glycemic gap might represent acute physiological stress due to AHF and found that BNP levels correlated positively with glycemic gap levels. Furthermore, we revealed that incorporating glycemic gap with BNP levels could further increase the discriminative powers of BNP levels to predict in-hospital mortality in AHF patients with diabetes. Serum albumin has many physiological properties, including anti-inflammatory, antioxidant, antiplatelet and colloid osmotic effects^52,53^. The low plasma oncotic pressure related to hypoalbuminemia can lead to a fluid shift from the intravascular to the interstitial space and facilitate acute pulmonary edema in patients with AHF. Hypoalbuminemia was associated with higher NYHA class and BNP levels and served as an independent prognostic factor for predicting in-hospital death for elderly patients with AHF^54,55^. Our findings are consistent with previous studies, and serum albumin levels remain a strong and independent predictor of in-hospital mortality in diabetic patients with AHF even after adjusting for BNP levels and age.

### Limitations

This study has a number of limitations. First, it was a single-center, retrospective investigation and may introduce selection bias. A prospective study using glycemic gap should be conducted in patients both with and without diabetes for further clarification. Second, the sufficiency of glycemic control while in hospital and the use of antidiabetic drugs might have interfered the outcomes. In the current study, we did not specifically investigate the influences of glycemic control during hospitalization. Intensive glucose-lowering therapy has no significant effect on the incidence of cardiac mortality or HF in patients with diabetes^56^. Marik *et al*. demonstrated that tight glycemic control does not remedy health care outcomes^57^. Third, insulin resistance or hyperinsulinemia is an independent predictor for AHF in cases of diabetes^58,59^. Several studies have reported that elevated leptin levels and reduction of adiponectin predict acute cardiovascular events^60,61^ and that the leptin/adiponectin ratio correlates well with insulin resistance in patients with diabetes^62^. Since the serum levels of insulin, leptin and adiponectin were not routinely measured in AHF patients, we could not clarify the relationship between glycemic gap and insulin resistance. Further prospective studies to incorporate serum insulin levels and leptin/adiponectin ratios into the glycemic gap may attain a clearer perception of the pathophysiology of SIH in diabetic patients with AHF.

## Conclusion

Elevated glycemic gap was an independent predictor of in-hospital mortality and ARF. The levels of glycemic gap might be used to estimate the severity of critical illness and clinical outcomes in patients with diabetes presenting with AHF.
